# The Effects of Flaxseed Consumption on Frailty and Sarcopenia in Patients With Liver Cirrhosis: An Open‐Labeled Randomized Controlled Clinical Trial

**DOI:** 10.1002/fsn3.71397

**Published:** 2025-12-29

**Authors:** Fereshteh Pashayee‐Khamene, Azita Hekmatdoost, Fatemeh Haidari, Amir Anushiravani, Behzad Hatami, Amir Sadeghi, Kambiz Ahmadi‐engali, Bizhan Helli

**Affiliations:** ^1^ Student Research Committee Ahvaz Jundishapur University of Medical Sciences Ahvaz Iran; ^2^ Nutrition and Metabolic Disease Research Center, Clinical Sciences Research Institute Ahvaz Jundishapur University of Medical Sciences Ahvaz Iran; ^3^ Department of Clinical Nutrition and Dietetics, Faculty of Nutrition Sciences and Food Technology, National Nutrition and Food Technology Research Institute Shahid Beheshti University of Medical Sciences Tehran Iran; ^4^ School of Health, Medical and Applied Sciences CQUniversity Brisbane Australia; ^5^ Digestive Oncology Research Center, Digestive Diseases Research Institute Tehran University of Medical Sciences Tehran Iran; ^6^ Gastroenterology and Liver Diseases Research Center, Research Institute for Gastroenterology and Liver Diseases Shahid Beheshti University of Medical Sciences Tehran Iran; ^7^ Department of Statistics and Epidemiology, Faculty of Public Health Ahvaz Jundishapur University of Medical Sciences Ahvaz Iran; ^8^ Department of Nutrition, School of Allied Medical Sciences Ahvaz Jundishapur University of Medical Sciences Ahvaz Iran

**Keywords:** body composition, flaxseed, frailty, liver cirrhosis, sarcopenia

## Abstract

Frailty and sarcopenia are common among patients with liver cirrhosis (LC), which can lead to a higher risk of several morbidities and mortality. The primary goal of this study was to explore the impact of flaxseed consumption on body composition, frailty and sarcopenia, as well as disease severity and inflammation in these patients. In this 12‐week, open‐labeled randomized clinical trial, we recruited fifty patients with LC who were randomly allocated to receive either flaxseed (30 g/d flaxseed powder) or no intervention. Body composition and anthropometric parameters, high‐sensitive C‐reactive protein (hs‐CRP), the model for end‐stage liver disease‐Na (MELD‐Na), frailty, sarcopenia, and muscle strength were assessed at the initiation and upon completion of the trial. After the study was completed, percent of body fat (PBF) and waist circumference (WC) decreased significantly in the flaxseed group when compared to the group's pre‐intervention values (*p* = 0.001 and *p* = 0.03, respectively). No significant changes were found regarding other body composition parameters (*p* > 0.05). MELD‐Na (*p* = 0.01) and hs‐CRP (*p* = 0.006) significantly declined only in the flaxseed group in comparison to the baseline. Also, a significant amelioration was found in liver frailty index (LFI) (*p* = 0.001), SARC‐F (*p* = 0.002), and hand grip (HG) (*p* = 0.008) as sarcopenia predictors after 12 weeks of intervention in the flaxseed group. These findings revealed that flaxseed consumption may positively affect muscle strength, reducing frailty and sarcopenia in individuals with cirrhosis by mitigating inflammation and lessening the severity of the disease.

**Trial Registration:** irct.ir registration number: IRCT20230514058189N1

## Graphical abstract

Flaxseed consumption can have favorable effects on muscle strength, reducing frailty and sarcopenia in patients with liver cirrhosis by alleviating inflammation and disease severity. The antioxidant and anti‐inflammatory effects of flaxseed may provide potential benefits as an adjunctive therapy for patients with cirrhosis.

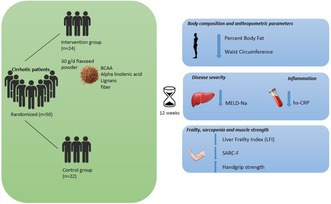

## 1 Introduction

Liver cirrhosis (LC) represents the end stage of chronic liver disorders, primarily caused by viral infections, autoimmune diseases, and steatohepatitis (Wiegand and Berg 2013). According to the Global Burden of Disease (GBD) report, 1.47 million deaths were attributed to liver cirrhosis worldwide in 2019. In Iran, cirrhosis accounted for 1.42% of all deaths in 2017, based on the most recent available statistics (Anushiravani and Ghajarieh Sepanlou 2019; Huang et al. 2023).

Frailty, sarcopenia, physical impairment, and changes in lean body mass are common and significant complications in patients with LC. These conditions are linked to a heightened risk of both morbidity and mortality (Bot et al. 2021; Figueiredo et al. 2005). In individuals with liver cirrhosis, the prevalence of sarcopenia and frailty ranges from 40% to 70% and 18% to 43%, respectively, depending on patient characteristics, evaluation techniques, and diagnostic criteria (Tandon et al. 2021).

The inflammatory condition in LC, characterized by elevated levels of proinflammatory factors, can trigger sarcopenia in multiple ways, such as by decreasing appetite and increasing hypermetabolism (Bojko 2019). Therefore, targeting inflammation in frailty management could be a beneficial approach to preventing the progression of frailty (Dalle et al. 2017). Despite consideration of a few new treatments that may help control frailty in LC, including hormone therapy, regular moderate exercise, adequate nutritional intake, and branched‐chain amino acid (BCAA) supplementation, there is no standard approach for prevention and treatment due to the multifactorial pathogenesis of frailty and the lack of comprehensive knowledge about its initiating factors (Bunchorntavakul and Reddy 2020; Tandon et al. 2021). However, because of the dynamic nature and potential reversibility of sarcopenia and frailty, the advancement of new and effective nutritional interventions to slow the progression of these complications in cirrhotic patients is needed (Tandon et al. 2021; Bunchorntavakul and Reddy 2020). Furthermore, there is increasing evidence of interest in using complementary medicine among patients with chronic liver diseases (Philips et al. 2024).

In recent years, flaxseed ( *Linum usitatissimum* ) has gained particular importance in the diet because of its nutraceutical properties and potential health benefits. Due to its functional components, such as branched‐chain amino acids, omega‐3 (alpha‐linolenic acid), lignans, and fiber, flaxseed could be considered a promising intervention as an adjuvant to help LC patients reduce sarcopenia and frailty (Shayan et al. 2020; Yari et al. 2016). Flaxseed protein, with its high content of branched‐chain amino acids, a favorable Fischer ratio (branched‐chain amino acids/aromatic amino acids), and a significant amount of cysteine as an antioxidant, can provide a desirable formulation for LC (Kajla et al. 2015). BCAAs may help maintain the liver‐muscle axis by stimulating protein synthesis, inhibiting proteolysis, and promoting autophagy (Holeček 2017; Tsien et al. 2015). Furthermore, flaxseed, as a potent inhibitor of pro‐inflammatory mediators, could be utilized as a novel anti‐inflammatory therapy (Kajla et al. 2015). Omega‐3, which is found abundantly in flaxseed, might serve as a therapeutic agent for preventing sarcopenia due to its anabolic effect on skeletal muscle (Abdelhamid et al. 2018; Robinson et al. 2018). In light of this, this randomized clinical trial aimed to investigate how a 12‐week flaxseed intervention impacts body composition, anthropometric parameters, inflammation, disease severity, frailty, sarcopenia, and muscle strength in patients with liver cirrhosis.

## 2 Methods and Materials

### 2.1 Study Design and Participants

A randomized, parallel‐controlled, open‐label clinical trial was performed in patients with LC. Sample size estimation was performed using the relevant formula, considering a type I error rate of 5% (*α* = 0.05) and a type II error rate of 20% (*β* = 0.20). We calculated the minimum sample size based on the model for end‐stage liver disease (MELD) score, one of the key outcomes (Nouri‐Vaskeh et al. 2020). Calculating the minimum sample size was defined on a 2.7 units difference in the mean model for end‐stage liver disease score with a power of 80% (*β* = 20%) leading to a sample size of 22 participants in each group. To account for potential dropouts, 25 subjects were eventually included in each group. In our trial, adult subjects with a diagnosis of cirrhosis who were referred to Taleghani and Shariati Hospital were assessed based on inclusion and exclusion criteria, and finally, 50 eligible patients were selected for participation. The inclusion criteria were being 18 to 75 years of age and having a confirmed diagnosis of LC by a gastroenterologist, a body mass index (BMI) between 18.5 and 30, no other organ failure, no history of flaxseed allergy, no consumption of consistently high amounts of nuts during the past 3 months, and no use of antioxidant supplements. The exclusion criteria included pregnancy or lactation, weight loss of more than 10%, following special diets, and consuming less than 80% of the total amount of flaxseed. The study was officially registered in the Iranian Registry of Clinical Trials. Informed consent was obtained from each participant after the study protocol was explained to them. The research was approved by the Ethics Committee of Ahvaz Jundishapour University of Medical Sciences (IR.AJUMS.REC.1402.090). This study was performed according to CONSORT reporting guidelines (Schulz et al. 2010).

### 2.2 Randomization

For randomization, the website “https://www.sealedenvelope.com/” was used, and randomization was performed using codes that were arranged in blocks of 4. To conceal the allocation sequence, the codes were placed in sealed envelopes, ensuring that neither the individuals nor the researcher was aware of the order of the codes or the assignment of participants to the intervention or control group.

### 2.3 Intervention and Follow‐Up

Patients in the intervention group received 30 g of flaxseed powder daily, while the control group did not receive any intervention. All subjects were visited every 4 weeks, and a 4‐week supply of flaxseed was provided to the intervention group until the end of the trial. Subjects were instructed to take the flaxseed powder with cold water on an empty stomach every day. Compliance with the study protocol was monitored by measuring the amount of returned flaxseed, and a loss of more than 20% of the provided milled flaxseed was considered an exclusion criterion.

### 2.4 Dietary Intake and Level of Physical Activity

Dietary records for 3 days (one weekend day and two weekdays) were collected at the beginning and end of the trial to assess total energy and macronutrient intake. The dietary information was analyzed using Nutritionist 4 software (N Squared Computing, San Bruno, CA, USA). The nutritional composition of flaxseed (including energy, macronutrients, and other components) was not incorporated into the patients' daily dietary intake analysis. Levels of physical activity were measured using the International Physical Activity Questionnaire (IPAQ), which has been validated among the Iranian population, both before and after the completion of the study (Moghaddam et al. 2012).

### 2.5 Body Composition and Anthropometric Parameters

Body composition and anthropometric parameters were assessed at weeks 0 and 12. Ascites status was assessed to ensure that fluid retention did not affect the anthropometric measurements. Prior to enrollment, the presence of ascites was assessed by a physician, and patients showing any signs of ascites were not included in the trial. Height, waist circumference (WC), and hip circumference were measured using a non‐stretchable measuring tape. The waist‐to‐hip ratio (WHR) was calculated by measuring the narrowest point of the waist and the widest point of the hips. Other parameters, such as weight, body mass index (BMI), lean body mass (LBM), soft lean mass (SLM), percent body fat (PBF), skeletal muscle mass (SMM), visceral fat level (VFL), visceral fat area (VFA), abdominal circumference (AC), and mass of body fat (MBF), were measured using the body composition analyzer X‐CONTACT 365 (Jawon Medical Co. Ltd., Korea). Mid‐arm circumference (MAC) was measured on the right arm to the nearest centimeter with the subject in an upright posture, arm flexed at 90°, at the midpoint between the olecranon and acromion (Jones 2004). Triceps skin fold (TSF) was measured to the nearest millimeter using a caliper (Holtain Ltd., Crymych, UK). The mid‐arm muscle circumference (MAMC) was calculated as MAC − (TSF × 3.1415) (Tartari et al. 2013). Peripheral fluid retention and ascites were assessed before anthropometric measurements. In addition, compared to body mass index (BMI), skinfolds are less influenced by fluid imbalance and may be more appropriate indicators during ascites or edema. According to the literature, mid‐arm circumference, triceps skin fold, and mid‐arm muscle circumference are effective measures for assessing body composition in cirrhotic patients (Morgan et al. 2006; Romeiro and Augusti 2015; Santos et al. 2019; Tandon et al. 2016). All anthropometric assessments were conducted by a third person who was not involved in the intervention.

### 2.6 Inflammation

To assess inflammation in patients, a 10 mL blood sample was obtained from each subject after a 12‐h fasting period. After centrifugation at 3000 rpm for 10 min, serum samples were transferred to microtubes and frozen at −70°C. The ELISA technique was used to measure high‐sensitivity C‐reactive protein (hs‐CRP), and the LDN Human CRP ELISA Kit (96 t, Germany) was applied.

### 2.7 Disease Severity

The Model for End‐Stage Liver Disease with Sodium (MELD‐Na) and Child–Pugh scores were used to determine the severity of liver cirrhosis. Laboratory values derived from blood samples, measured by an automated analyzer, included serum creatinine, serum total bilirubin, serum sodium, and the international normalized ratio (INR), which were used to calculate the MELD‐Na score. For the calculation of the Child–Pugh score, we used serum bilirubin, serum albumin, INR, and the presence of ascites and encephalopathy (Puentes et al. 2018).

### 2.8 Frailty, Sarcopenia, and Muscle Strength Assessment

Handgrip (HG) strength was measured using a hand dynamometer (CAMRY Electronic Hand Dynamometer EH101). Measurements were taken while the subject was seated with their back straight and elbows bent at 90 degrees. The subject was instructed to grip the handle tightly for 3 s. Three successive tests were performed, and the average handgrip strength was recorded (Song et al. 2021) following the recommendations of the European Working Group on Sarcopenia in Older People (EWGSOP), the cut‐off points for low muscle strength are < 28 kg for males and < 18 kg for females (Chen et al. 2020). Frailty was evaluated using the Liver Frailty Index (LFI). Given that the LFI was specifically designed for cirrhotic patients, it was used as the reference tool for diagnosing frailty (Lai et al. 2017). The LFI comprises three performance‐based tests: (1) grip strength, determined by averaging three trials with the dominant hand; (2) time to complete five consecutive chair rises with arms folded across the chest; and (3) a balance test, which measures the time to balance in three positions (feet placed side‐by‐side, semi‐tandem, and tandem) for a maximum of 10 s each. The LFI is calculated based on the results of these tests, using a calculator available at http://liverfrailtyindex.ucsf.edu. The LFI categories are as follows: LFI < 3.2: Robust, 3.3 ≤ LFI ≤ 4.4: Pre‐Frail, and LFI > 4.5: Frail (Lai et al. 2021). Additionally, the SARC‐F questionnaire was used to assess health status changes related to sarcopenia. The SARC‐F questionnaire contains five questions: strength, assistance with walking, rising from a chair, climbing stairs, and falls. Each component is scored from 0 to 2 points, with total scores ranging from 0 to 10. A score of 4 or more indicates a high risk of sarcopenia and potential adverse outcomes (Malmstrom et al. 2016).

### 2.9 Statistical Analysis

The analysis of the trial data was performed using the Statistical Package for the Social Sciences (SPSS Inc., Chicago, IL, USA, version 21.0). Normality of the variables was assessed using the Kolmogorov–Smirnov test. Categorical variables are presented as frequencies (percentages), while quantitative variables are presented as means ± standard deviations (SD). To assess differences between groups at both time points, the unpaired *t*‐test was applied. To compare mean changes within groups from pretest to posttest, the paired *t*‐test was performed. To evaluate the effectiveness of the intervention on significant changes in variables, analysis of covariance (ANCOVA) was conducted. The ANCOVA model included baseline values of each variable, as well as mean changes in calorie intake, physical activity, BMI, age, alcohol consumption, smoking status, zinc and other supplements, and the presence of underlying diseases as confounding factors. All statistical analyses were performed by a third person who was not engaged in the intervention. A *p*‐value of < 0.05 was considered statistically significant.

## 3 Results

### 3.1 Subjects

**FIGURE 1:**
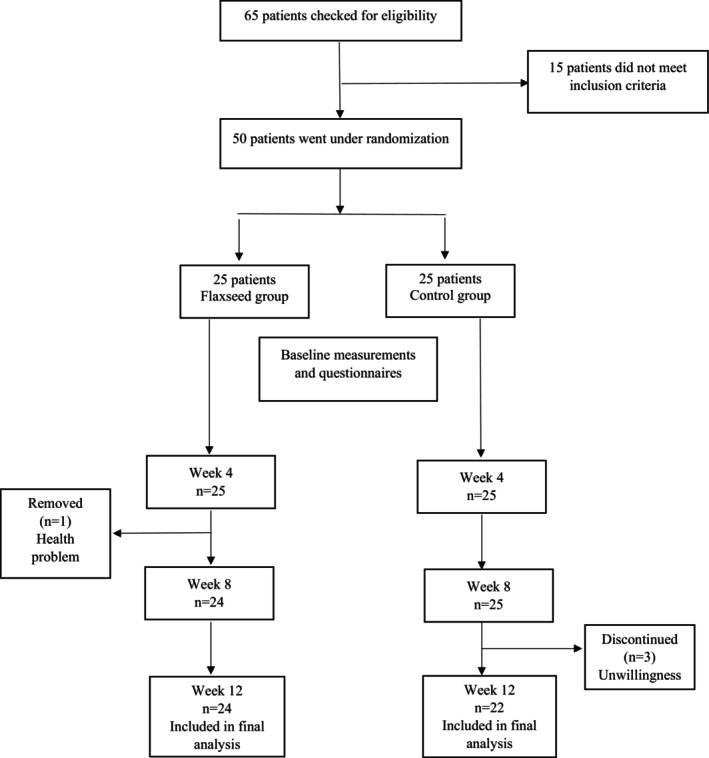
Study flow chart.

Out of 65 patients assessed for eligibility based on the inclusion and exclusion criteria, 50 were selected for enrollment. Among the eligible participants who were randomly assigned to each group, 3 patients (due to unwillingness) in the control group and 1 patient (due to a health problem) in the flaxseed group failed to complete the study. Consequently, 46 patients were included in the final analysis, as shown in Figure 1.

Baseline characteristics of the subjects are presented in Table 1. Regarding other initial characteristics, 39.2% of participants were smokers, 17.3% had a history of alcohol consumption, 8.6% were taking zinc supplements, and 41.3% used other types of supplements. Additionally, 28.3% of subjects did not have any other underlying diseases, while the remaining participants had concurrent diabetes or cardiovascular diseases alongside cirrhosis. The majority of patients (78.3%) were categorized as Class A based on the Child–Pugh score. Concerning the etiology of liver cirrhosis, 39.2% of patients had fatty liver disease, and 23.9% had viral hepatitis. There were no significant differences in these baseline characteristics between the two groups.

**TABLE 1:** Baseline characteristics of patients with LC by treatment groups.

Variables	Control group (*n* = 22)	Flaxseed group (*n* = 24)	*p*
Age (years)	56.23 ± 15.09	52.17 ± 12.98	0.33
Sex			
Male	11 (23.9)	17 (37)	0.22
Female	11 (23.9)	7 (15.2)	
Weight (kg)	75.13 ± 11.82	73.67 ± 15.82	0.72
BMI (kg/m^2^)	27 ± 2.66	26.05 ± 3.93	0.35
Energy intake (kcal/day)	1374.17 ± 336.47	1668.70 ± 482.75	**0.02**
Physical activity (MET‐min/w)	960.47 ± 1723.85	1073.25 ± 1877.85	0.83

*Note:* Variables are shown as mean ± SD, number of participants and percentages. *p* value: independent sample *t*‐test. Bold values indicate statistically significant results (*p* < 0.05).

### 3.2 Dietary Analysis and Physical Activity

The energy, dietary intake, and physical activity information are shown in Table 2. In participants assigned to the flaxseed group, there was an increase in protein intake (92.85 ± 29.70 vs. 95.96 ± 30.55, *p* = 0.05) and fiber intake (12.47 ± 5.22 vs. 13.41 ± 5.02, *p* = 0.01) after 12 weeks compared to baseline. At the end of the trial, fiber intake also increased in the control group (10.30 ± 3.64 vs. 11.58 ± 3.62, *p* = 0.005). Comparison of total energy intake between groups showed significant differences at both time points between the flaxseed and control groups (1668.70 ± 482.75 vs. 1374.17 ± 336.47, *p* = 0.02 and 1685.01 ± 476.25 vs. 1403.98 ± 331.57, *p* = 0.02, respectively). Comparisons of other macronutrient intake and physical activity, both within and between groups, did not show any significant differences.

**TABLE 2:** Mean changes (SD) from baseline in calories, macronutrient, and physical activity by treatment groups.

Variables	Groups	Baseline	After 12 weeks	Changes	*p* ^a^
Total energy (kcal/day)	Flaxseed	1668.70 ± 482.75	1685.01 ± 476.25	16.30 ± 202.05	0.69
	Control	1374.17 ± 336.47	1403.98 ± 331.57	29.80 ± 173.82	0.43
	*p* ^b^	**0.02**	**0.02**	0.81	
Total energy (kcal/kg)	Flaxseed	23.59 ± 8.16	24.00 ± 8.77	0.41 ± 2.97	0.50
	control	18.83 ± 6.02	19.12 ± 5.64	0.28 ± 2.80	0.63
	*p*	**0.03**	**0.02**	0.88	
Carbohydrate (g/day)	Flaxseed	204.80 ± 67.24	203.59 ± 66.94	−1.21 ± 39.02	0.88
	Control	192.79 ± 64.83	196.68 ± 68.00	3.88 ± 40.06	0.65
	*p*	0.54	0.73	0.66	
Protein (g/day)	Flaxseed	92.85 ± 29.70	95.96 ± 30.55	3.11 ± 7.36	**0.05**
	Control	79.28 ± 21.06	83.65 ± 25.28	4.37 ± 14.29	0.16
	*p*	0.08	0.14	0.54	
Protein (g/kg)	Flaxseed	1.31 ± 0.45	1.36 ± 0.50	0.04 ± 0.11	**0.05**
	Control	1.07 ± 0.31	1.12 ± 0.34	0.05 ± 0.17	0.17
	*p*	**0.04**	0.07	0.9	
Fat (g/day)	Flaxseed	43.10 ± 14.19	43.04 ± 16.84	1.00 ± 5.14	0.29
	Control	40.66 ± 11.92	39.62 ± 13.53	0.07 ± 8.95	0.97
	*p*	0.54	0.46	0.61	
Fiber (g/day)	Flaxseed	12.47 ± 5.22	13.41 ± 5.02	0.94 ± 1.77	**0.01**
	Control	10.30 ± 3.64	11.58 ± 3.62	1.27 ± 1.88	**0.005**
	*p*	0.11	0.16	0.55	
Physical activity (MET‐min/w)	Flaxseed	1073.25 ± 1877.85	1025.64 ± 1883.29	−47.60 ± 175.81	0.20
	Control	960.47 ± 1723.85	1013.42 ± 1798.55	−16.70 ± 109.27	0.50
	*p*	0.83	0.98	0.5	

*Note:* All variables are shown as mean ± SD. Bold values indicate statistically significant results (*p* < 0.05).

^a^ Paired sample *t*‐test.

^b^ Independent sample *t*‐test.

### 3.3 Body Composition and Anthropometric Parameters

As shown in Table 3, a significant decline in percent body fat was observed only in the intervention group at the end of week 12 compared to baseline (27.07 ± 7.55 vs. 25.20 ± 8.17, *p* = 0.001). Additionally, a significant difference in percent body fat between groups was noted after 12 weeks (flaxseed group: 25.20 ± 8.17 vs. control group: 29.96 ± 7.56, *p* = 0.04). Waist circumference also decreased in the intervention group by −2.90 ± 4.03 cm at week 12, which was significant (*p* = 0.03). Other anthropometric parameters did not show significant changes.

**TABLE 3:** Body composition and anthropometric parameters, inflammation and disease severity and their changes by treatment groups at baseline and at the end of the trial.

Variables	Groups	Baseline	After 12 weeks	*p* ^a^	changes	*p* ^c^
**Body composition and anthropometric parameters**						
Weight (kg)	Flaxseed	73.67 ± 15.82	73.87 ± 15.31	0.68	0.20 ± 2.40	
	Control	75.13 ± 11.82	75.34 ± 12.21	0.72	0.20 ± 2.73	
	*p* ^b^	0.72	0.72		0.99	
Body mass index (kg/m^2^)	Flaxseed	26.05 ± 3.93	26.13 ± 3.78	0.73	0.07 ± 0.98	
	Control	27 ± 2.66	27.06 ± 2.76	0.76	0.06 ± 0.93	
	*p*	0.35	0.34		0.97	
Lean body mass (kg)	Flaxseed	53.42 ± 11.54	54.16 ± 11.95	0.24	0.73 ± 30.0	
	Control	52.43 ± 10.51	52.76 ± 11.12	0.65	0.33 ± 3.46	
	*p*	0.76	0.68		0.67	
Soft lean mass (kg)	Flaxseed	49.24 ± 10.77	49.83 ± 11.12	0.35	0.58 ± 3.02	
	Control	48.14 ± 9.90	48.50 ± 10.50	0.61	0.36 ± 3.33	
	*p*	0.72	0.68		0.81	
Skeletal muscle mass (kg)	Flaxseed	29.50 ± 6.45	29.90 ± 6.70	0.27	0.40 ± 1.76	
	Control	28.83 ± 5.92	28.97 ± 6.26	0.76	0.13 ± 2.10	
	*p*	0.71	0.62		0.64	
Percent body fat	Flaxseed	27.07 ± 7.55	25.20 ± 8.17	**0.001**	−1.87 ± 2.45	
	Control	30.28 ± 7.24	29.96 ± 7.56	0.71	−0.31 ± 4.09	
	*p*	0.14	**0.04**		0.12	
Visceral fat level	Flaxseed	12.12 ± 3.67	12.04 ± 3.53	0.81	−0.08 ± 1.71	
	Control	12.54 ± 3.30	12.27 ± 2.91	0.56	−0.27 ± 2.18	
	*p*	0.68	0.81		0.74	
Visceral fat area	Flaxseed	129.87 ± 64.00	127.58 ± 60.47	0.65	−2.29 ± 24.90	
	Control	125.27 ± 57.29	118.09 ± 45.79	0.28	−7.18 ± 30.51	
	*p*	0.79	0.55		0.55	
Waist circumference (cm)	Flaxseed	98.45 ± 12.54	95.85 ± 13.27	**0.03**	−2.90 ± 4.03	
	Control	99.55 ± 11.59	101.85 ± 11.24	0.76	0.33 ± 3.16	
	*p*	0.46	0.13		0.06	
Abdominal circumference (cm)	Flaxseed	89.32 ± 10.59	88.11 ± 9.15	0.14	−1.20 ± 3.90	
	Control	89.25 ± 8.48	88.90 ± 8.30	0.73	−0.34 ± 4.69	
	*p*	0.97	0.76		0.49	
Waist to hip ratio	Flaxseed	0.95 ± 0.05	0.90 ± 0.8	0.08	−0.03 ± 0.06	
	Control	0.93 ± 0.07	0.91 ± 0.06	0.28	−0.01 ± 0.05	
	*p*	0.35	0.51		0.47	
Mass of body fat	Flaxseed	19.69 ± 8.36	20.39 ± 7.41	0.25	0.69 ± 2.93	
	Control	22.71 ± 6.13	22.38 ± 6.37	0.65	−0.33 ± 3.38	
	*p*	0.17	0.33		0.27	
Mid‐arm circumference (cm)	Flaxseed	30.79 ± 4.20	30.79 ± 3.78	1.00	00.00 ± 1.23	
	Control	31.29 ± 4.01	31.02 ± 3.32	0.36	−0.27 ± 1.36	
	*p*	0.68	0.82		0.48	
Triceps skin fold (mm)	Flaxseed	15.19 ± 8.32	16.35 ± 13.67	0.63	1.15 ± 11.78	
	Control	11.41 ± 5.52	10.70 ± 5.20	0.28	−0.70 ± 3.00	
	*p*	0.08	0.07		0.46	
Mid arm muscle circumference; (cm)	Flaxseed	26.63 ± 3.51	27.25 ± 3.41	0.14	0.62 ± 2.01	
	Control	27.71 ± 3.94	27.56 ± 3.30	0.66	−0.15 ± 1.60	
	*p*	0.32	0.75		0.16	
**Inflammation**						
High‐sensitive C reactive protein (ng/mL)	Flaxseed	4880.94 ± 4322.46	3794.56 ± 4030.87	**0.006**	−1086.38 ± 1753.01	
	Control	3933.91 ± 3070.01	5560.74 ± 6747.96	0.20	1626.82 ± 5823.94	
	*p*	0.39	0.28		**0.03**	**0.02**
**Disease severity**						
MELD‐Na	Flaxseed	9.25 ± 3.46	7.66 ± 2.21	**0.01**	−1.58 ± 2.78	
	Control	9.95 ± 3.52	10.81 ± 4.72	0.06	0.86 ± 2.07	
	*p*	0.49	**0.008**		**0.002**	**< 0.001**

*Note:* All variables are shown as mean ± SD. Bold values indicate statistically significant results (*p* < 0.05).

Abbreviation: MELD‐Na, Model for End‐Stage Liver Disease‐Na.

^a^ Paired sample *t*‐test.

^b^ Independent sample *t*‐test.

^c^ ANCOVA's model, adjusted for the baseline value of each variable, mean changes in calorie intake, physical activity, and BMI, in addition to age, alcohol drinking, smoking status, zinc, and other kinds of supplements and presence of underlying diseases; as an indication to study the comparison of the mean changes related to each variable between the two groups before and after intervention.

### 3.4 Inflammation and Disease Severity

According to Table 3, in the flaxseed group, a significant decrease in high‐sensitive C‐reactive protein (hs‐CRP) was observed from baseline to the end of the study (4880.94 ± 4322.46 vs. 3794.56 ± 4030.87, *p* = 0.006). Importantly, the analysis of covariance indicated that hs‐CRP (*p* = 0.02) decreased significantly after flaxseed consumption compared to the control group.

A significant reduction in the MELD‐Na score was observed in the flaxseed group by the end of the study compared to baseline (9.25 ± 3.46 vs. 7.66 ± 2.21, *p* = 0.01). The difference between changes remained significant after adjusting for all covariates (*p* < 0.001). Regarding the Child–Pugh score, before consumption in the flaxseed group, 79.2% and 4.2% of patients were classified as Category A and C, respectively, while after the study these categories changed to 95.8% and 0% (data not shown).

### 3.5 Frailty, Sarcopenia and Muscle Strength Assessment

Table 4 shows a significant decline in the Liver Frailty Index (LFI) in the flaxseed group after 12 weeks (*p* < 0.001). Additionally, a significant difference in LFI was found between the two groups at the end of the study (*p* = 0.008). We observed a significant improvement in SARC‐F in the post‐intervention period compared to the pre‐intervention period in the flaxseed group (*p* = 0.008). According to the analysis of covariance, SARC‐F declined significantly after flaxseed intake compared to the control group (*p* = 0.007).

**TABLE 4:** Frailty, sarcopenia and muscle strength assessments and their changes by treatment groups at baseline and at the end of the trial.

Variables	Groups	Baseline	After 12 weeks	*p* ^a^	Changes	*p* ^c^
LFI	Flaxseed	4.04 ± 0.59	3.79 ± 0.58	**< 0.001**	−0.25 ± 0.27	
	Control	4.07 ± 0.60	4.17 ± 0.79	0.25	0.09 ± 0.39	
	*p* ^b^	0.86	0.06		**0.001**	**0.008**
SARCF	Flaxseed	1.75 ± 1.89	1.12 ± 1.75	**0.008**	−0.62 ± 1.05	
	Control	1.77 ± 1.68	2.45 ± 2.68	0.06	0.68 ± 1.64	
	*p*	0.96	0.057		**0.002**	**0.007**
HG (kg)	Flaxseed	26.78 ± 11.39	27.92 ± 10.84	0.20	1.13 ± 4.26	
	Control	25.40 ± 12.20	23.11 ± 10.65	**0.01**	−2.28 ± 4.07	
	*p*	0.69	0.13		**0.008**	**0.05**

*Note:* All variables are shown as mean ± SD. Bold values indicate statistically significant results (*p* < 0.05).

Abbreviations: HG, Hand grip; LFI, Liver frailty index; SARC‐F, Strength, Ambulation, Rising from a chair, Stair climbing and history of Falling.

^a^ Paired sample *t*‐test.

^b^ Independent sample *t*‐test.

^c^ ANCOVA's model, adjusted for the baseline value of each variable, mean changes in calorie intake, physical activity, and BMI, in addition to age, alcohol drinking, smoking status, zinc, and other kinds of supplements and presence of underlying diseases; as an indication to study the comparison of the mean changes related to each variable between the two groups before and after intervention.

Regarding handgrip strength as a muscle strength parameter, there was no significant difference between the groups at both measurement times. However, a significant difference was noted in the change in handgrip strength between the flaxseed and control groups (1.13 ± 4.26 vs. −2.28 ± 4.07, *p* = 0.008). This difference remained marginally significant after adjusting for all covariates (*p* = 0.05). Additionally, the decline in handgrip strength in the control group after 12 weeks was significant (25.40 ± 12.20 vs. 23.11 ± 10.65, *p* = 0.01).

## 4 Discussion

To our knowledge, no previous clinical trial has assessed the impact of flaxseed powder on frailty and sarcopenia in cirrhotic patients. In this study, we found significant improvements in all frailty predictors, including the LFI, SARC‐F, and HG in patients with LC. It appears that flaxseed, as a functional food, may exert beneficial effects on muscle strength and physical performance in LC patients due to its considerable amount of BCAA (25 g per 100 g), a high Fischer ratio (4.7), and omega‐3 fatty acids (Ganorkar and Jain 2013; Oomah 2001). The advantages of both BCAA (leucine, isoleucine, and valine) and omega‐3 fatty acids on muscle strength and sarcopenia have been investigated in previous studies (Dupont et al. 2019; Ebadi et al. 2019; Meyer et al. 2020).

The surge of interest in the utility of BCAA in these patients is related to several advantages, such as increased nitrogen balance, better energy and protein intake, and improvements in serum albumin levels (Marchesini et al. 2003; Nakaya et al. 2007). Among BCAAs, leucine directly stimulates protein synthesis and decreases autophagy by activating mTORC1 (Dasarathy and Merli 2016). Similar to our investigation, Lattanzi et al. demonstrated the ameliorating effect of beta‐hydroxy‐beta‐methylbutyrate (HMB), a metabolite of leucine, on the LFI score in LC patients after 12 weeks of supplementation. However, HG, lean body mass, and severity of cirrhosis were not significantly modified during the study (Lattanzi et al. 2021). In contrast, another randomized trial involving 116 patients with LC found that BCAA supplementation did not improve HG over 56 weeks (Les et al. 2011). The differing results regarding HG in this study may be explained by the severity of hepatic failure and sarcopenia in these patients, who were hospitalized for 2 months with episodes of encephalopathy. Therefore, the initial level of sarcopenia might have influenced the final results.

Growing evidence has shown that omega‐3 fatty acids might have an anabolic effect on skeletal muscles. Thus, omega‐3 supplementation may be considered a promising agent for preventing the progression of sarcopenia with minimal adverse effects (Robinson et al. 2018). Recent randomized controlled trials (RCTs) have demonstrated that omega‐3 supplements can help stimulate muscle protein synthesis in the elderly. Smith et al. concluded that omega‐3 supplementation (daily intake of 1.86 g EPA and 1.5 g DHA) for 8 weeks in subjects aged ≥ 65 years can enhance muscle protein synthesis compared to corn oil supplementation (Smith et al. 2011). Consistent with our results, Robinson et al. found in a cohort study that each additional serving of fatty fish was associated with an increase of 0.43 kg in HG in men and 0.48 kg in women aged 59–73 years (Robinson et al. 2008). Although the precise mechanism by which inflammation causes sarcopenia remains unclear, there may be an opportunity to target sarcopenia by reducing inflammation with anti‐inflammatory agents like omega‐3 (Dupont et al. 2019). Inflammation during chronic diseases often leads to inappropriate muscle autophagy (Bojko 2019). Elevated levels of proinflammatory cytokines in plasma are proposed to play a key role in the progression of sarcopenia by affecting anabolic and catabolic signaling pathways (Bian et al. 2017). Increased activity of the ubiquitin‐proteasome pathway (UPP), induced by elevated proinflammatory cytokines, mediates muscle autophagy (Campos et al. 2018). Two other hypothetical mechanisms for these effects include the activation of mTOR and a reduction in insulin resistance. Yoshino et al. (2016) found that omega‐3 supplementation might induce gene expression changes that decrease the expression of inhibitory signaling pathways on mTOR, favoring skeletal muscle anabolism. On the other hand, due to the key role of insulin signaling in mTOR activation, some studies suggest that omega‐3 therapy may decrease insulin resistance (Albert et al. 2014). Conversely, Krzymińska‐Siemaszko et al. did not find an effect of omega‐3 supplementation on muscle mass or muscle strength after 12 weeks in older adults (daily intake of 660 mg EPA and 440 mg DHA) (Krzymińska‐Siemaszko et al. 2015). The conflicting results may be attributed to the smaller sample size and lower dosage of omega‐3 used in this study.

The reduction in hs‐CRP after 12 weeks of intervention supports the anti‐inflammatory effects of flaxseed consumption discussed earlier. Consistent with our findings, a meta‐analysis by Custodero et al. (2018) reported a reduction in CRP levels following omega‐3 supplementation in older adults. The anti‐inflammatory properties of omega‐3 are attributed to various mechanisms, including the modification of phospholipid fatty acid composition in cell membranes, disruption of lipid rafts, inhibition of nuclear factor kappa B (NF‐κB) activation—which decreases the expression of inflammatory genes—activation of peroxisome proliferator‐activated receptor γ (PPARγ), an anti‐inflammatory transcription factor, and binding to the G protein‐coupled receptor GPR120 (Calder 2015). Therefore, our results regarding the changes in hs‐CRP levels and frailty assessments seem reasonable, as inflammation and sarcopenia are closely related, as also indicated by other studies. Bano et al. (2017) reported in a meta‐analysis that sarcopenia is associated with increased CRP levels in serum. Furthermore, other studies have identified higher CRP levels as an important risk factor for muscle strength loss (Schaap et al. 2006). Similarly, in our study, we observed a significant improvement in HG as a muscle strength indicator, along with a reduction in hs‐CRP levels. In contrast, Smith et al. did not find a significant effect on CRP levels (Smith et al. 2011). A plausible reason for this lack of significance may be the selection of healthy subjects with lower levels of inflammatory markers in their study.

We found a significant ameliorative effect of flaxseed consumption on MELD‐Na in the current investigation. Higher levels of circulating pro‐inflammatory cytokines are correlated with disease severity, and overwhelming evidence shows that systemic inflammation plays a major role in the natural progression of cirrhosis (Engelmann et al. 2021). The significant reduction in hs‐CRP levels observed in this study could align with the observed alleviation in disease severity. Zhu et al. (2022) reported an association between elevated serum hs‐CRP levels and the severity of steatosis and fibrosis. Additionally, consistent with our findings, several studies have revealed a correlation between sarcopenia and Child–Pugh or MELD scores (Montano‐Loza et al. 2014; Tandon et al. 2012). Montano‐Loza et al. noted an association between sarcopenia and higher Child–Pugh scores (Jones 2004). Thus, the observed changes in all outcomes in our study appear justifiable.

We also observed a significant decline in PBF and waist circumference, which could be valuable for patients with LC. A recent meta‐analysis involving 2867 subjects revealed a significant reduction in waist circumference following flaxseed supplementation (Musazadeh et al. 2024). Additionally, a study found that consuming 40 g of flaxseed for 12 weeks significantly decreased fat mass among perimenopausal overweight women (Aguilar et al. 2017). The anti‐obesity effects of flaxseed can be attributed to its various compounds. Alpha‐linolenic acid (ALA), soluble and insoluble fibers, and lignans are potential anti‐obesity elements in flaxseed (Kajla et al. 2015). ALA can be converted into eicosapentaenoic acid (EPA) and docosahexaenoic acid (DHA) in humans. In vivo research suggests that EPA and DHA may reduce obesity and decrease adipogenesis by overexpressing peroxisome proliferator‐activated receptor‐gamma (PPAR‐γ) and downregulating G‐protein‐coupled receptor 120 (GPR‐120) (Wei et al. 2021). Furthermore, a high‐fiber diet, particularly one supplemented with flaxseed, can alter intestinal flora, delay stomach emptying, and increase the production of short‐chain fatty acids (SCFAs). However, the study by Shareghfarid et al. (2022) reported no significant effect of 2000 mg/day of flaxseed oil on body fat over 14 weeks. This lack of efficacy might be due to the use of flaxseed oil rather than flaxseed powder, which results in the elimination of flaxseed fiber.

Although we expected to find a significant increase in LBM and other muscle‐related parameters in our study, in line with sarcopenic parameters, we did not achieve these findings. A possible explanation for not observing a significant change may be the short duration of our trial. Therefore, further investigations with a longer duration are recommended.

A major strength of our study is that it represents the first randomized trial to investigate the effects of flaxseed in patients with liver cirrhosis focusing on sarcopenia. In accordance with methodological standards, a comprehensive and multidimensional approach to outcome assessment, statistically significant findings, and biological plausibility are other strengths of this investigation. However, the open‐label design and the absence of computed tomography (CT) or dual‐energy X‐ray absorptiometry (DXA)—which are considered gold standards for body composition assessment in LC—are important limitations of our research. In addition, the small sample size, which may have resulted in some statistical errors, the short intervention period, and lack of clinical endpoints (hospitalizations, quality of life, or mortality) due to short follow‐up duration are other limitations of our study. Although analyses were adjusted for energy intake, we cannot completely rule out the possibility that differences in dietary energy between groups may have contributed to the observed changes in LFI and MELD. Future studies with stricter dietary control to provide more accurate estimations of dietary intake and larger sample sizes in cirrhotic patients are warranted to confirm these findings.

## 5 Conclusion

We concluded that consuming 30 g of flaxseed powder per day can reduce disease severity, inflammation, WC, and PBF in patients with LC. We also observed that flaxseed consumption may exert positive effects on frailty, sarcopenia, and muscle strength. It appears that flaxseed consumption for 12 weeks has a greater impact on muscle strength than on muscle mass.

## Author Contributions

Conceptualization, F.P.‐K., B.H., A.H., F.H.; and F.H.; supervision, B.H., A.S., A.A., B.H.; formal analysis, K.A., P.F.; methodology, B.H., F.P.‐K., and A.H.; project administration, B.H., F.P.‐K. and A.H.; writing – original draft, F.P.‐K.; writing – review and editing, B.H. and A.H. All authors read and approved the final manuscript.

## Funding

This clinical trial was supported by a Grant from the Ahvaz Jundishapur University of Medical Sciences (Grant/Award number: NRC‐0202).

## Ethics Statement

The research was approved by the Ethics Committee of Ahvaz Jundishapour University of Medical Sciences (IR.AJUMS.REC.1402.090). The study protocol was conducted in accordance with the Declaration of Helsinki.

## Consent

Informed consent was obtained from each participant after the study protocol was explained to them.

## Conflicts of Interest

The authors declare no conflicts of interest.

## Data Availability

The data that support the findings of this study are available from the corresponding author upon reasonable request.
